# Blink-related EEG oscillations are neurophysiological indicators of subconcussive head impacts in female soccer players: a preliminary study

**DOI:** 10.3389/fnhum.2023.1208498

**Published:** 2023-07-19

**Authors:** Sahar Sattari, Rebecca Kenny, Careesa Chang Liu, Sujoy Ghosh Hajra, Guy A. Dumont, Naznin Virji-Babul

**Affiliations:** ^1^School of Biomedical Engineering, The University of British Columbia, Vancouver, BC, Canada; ^2^Department of Rehabilitation Sciences, The University of British Columbia, Vancouver, BC, Canada; ^3^Department of Biomedical Engineering and Science, Florida Institute of Technology, Melbourne, FL, United States; ^4^Department of Electrical and Computer Engineering, The University of British Columbia, Vancouver, BC, Canada; ^5^BC Children’s Hospital Research Institute, Vancouver, BC, Canada; ^6^Department of Physical Therapy, Djavad Mowafaghian Centre for Brain Health, The University of British Columbia, Vancouver, BC, Canada

**Keywords:** subconcussion, blink-related oscillations, spontaneous blinking, EEG, soccer heading, sports concussion

## Abstract

**Introduction:**

Repetitive subconcussive head impacts can lead to subtle neural changes and functional consequences on brain health. However, the objective assessment of these changes remains limited. Resting state blink-related oscillations (BROs), recently discovered neurological responses following spontaneous blinking, are explored in this study to evaluate changes in BRO responses in subconcussive head impacts.

**Methods:**

We collected 5-min resting-state electroencephalography (EEG) data from two cohorts of collegiate athletes who were engaged in contact sports (SC) or non-contact sports (HC). Video recordings of all on-field activities were conducted to determine the number of head impacts during games and practices in the SC group.

**Results:**

In both groups, we were able to detect a BRO response. Following one season of games and practice, we found a strong association between the number of head impacts sustained by the SC group and increases in delta and beta spectral power post-blink. There was also a significant difference between the two groups in the morphology of BRO responses, including decreased peak-to-peak amplitude of response over left parietal channels and differences in spectral power in delta and alpha frequency range post-blink.

**Discussion:**

Our preliminary results suggest that the BRO response may be a useful biomarker for detecting subtle neural changes resulting from repetitive head impacts. The clinical utility of this biomarker will need to be validated through further research with larger sample sizes, involving both male and female participants, using a longitudinal design.

## 1. Introduction

Traumatic brain injuries (TBI) sustained repeatedly throughout a person’s life may contribute to the development of neurodegenerative disorders (Lehman et al., 2012; Gardner et al., 2018; Mackay et al., 2019). While it is well documented in the literature that repeated mild traumatic brain injuries (also referred to as concussions) have significant negative consequences on brain health, there is increasing concern about subconcussive head impacts that do not result in a clinical diagnosis of concussion (Ntikas et al., 2022). While subconcussive head impacts do not necessarily cause concussion or evoke clinical symptoms, recent research is beginning to show that repeated subconcussive head impacts may result in behavioral and neurophysiological changes. Neuroimaging studies are revealing changes in both brain structure and function even before any neurocognitive symptoms are observed due to subconcussive head impacts (Ntikas et al., 2022). Behavioral studies have documented impaired cognition with changes in short- and long-term memory (Di Virgilio et al., 2016), learning (McAllister et al., 2012), attention (Wilson et al., 2015) as well as changes in sleep, social activities and activities of daily living (see Dioso et al., 2022) as a result of these impacts. Subconcussive and concussive head impacts pose a notable societal burden and economic strain on the healthcare system due to the often unmet challenges in occupational and educational performance (Dioso et al., 2022). Importantly, the lack of well-defined objective measures of the cumulative effects of sub-concussion and the resulting changes in behavior is one of the major challenges in the field.

Electroencephalography (EEG) has emerged as promising modalities in the diagnosis and classification of mTBI. Resting state EEG has revealed significant alterations in the functional organization of the brain in individuals with concussions with respect to healthy controls with key features being (1) changes in oscillatory power in beta, delta and theta frequency bands (Balkan et al., 2015) (2) changes in coherence and functional connectivity (Virji-Babul et al., 2014) and (3) disrupted information flow patterns (Hristopulos et al., 2019). In addition, our team has recently developed a deep learning algorithm that utilizes resting-state raw EEG data for the classification of concussions (Thanjavur et al., 2021). Furthermore, EEG-derived event-related potentials (ERP) have been employed for the detection of abnormalities associated with concussions and subconcussive head impacts (Moore et al., 2017; Fickling et al., 2019).

Resting-state functional brain connectivity alterations, particularly in the default mode network (DMN) have been observed in various functional magnetic resonance imaging (fMRI) studies. Johnson et al. observed connectivity changes in a cohort of male collegiate rugby players before and after a single game; which were also associated with previous concussions (Johnson et al., 2014). Abbas et al. (2015) confirmed this finding by showing hyperconnectivity within the DMN in a cohort of male high school football players compared to non-contact sports players.

Soccer is a sport that involves repetitive heading, with players using their unprotected head to manipulate the ball. There is growing concern over the cumulative impact of heading on the brain and the potential neurological consequences leading to recent changes in the rules to restrict heading exposure among children (Anna, 2020). Generally, soccer players sustain fewer concussions over the course of their careers compared with other contact sports such as rugby and hockey (Daneshvar et al., 2011; Zuckerman et al., 2015). This is generally attributed to the fact that sport-related exposure in soccer players is due to much lighter impact when heading the soccer ball, making them a suitable population for conducting studies on subconcussive head injuries without the confound of concussion.

Here we report on our efforts to address this challenge using a novel brain-based approach. Blink-related oscillations (BRO) are a recently discovered neurophysiological response following spontaneous blinking, and are distinct from the oculomotor effects of blinking (Liu et al., 2020). Although blinking has not been previously associated with cognition, findings from behavioral studies have pointed to a relationship between blink rate and cognitive processes such as attention and memory processing (Paprocki and Lenskiy, 2017). In line with such evidence, neuroimaging studies have characterized the brain response to spontaneous blinking as increased activity in the delta frequency band (0.5–4 Hz) occurring after blinking, and found that it is associated with activity in bilateral precuneus (Liu et al., 2017). This response was studied as a potential biomarker for evaluation of disorders of consciousness (Bonfiglio et al., 2013) and has been shown to reflect the level of cognitive load (Liu et al., 2019b) and environmental changes (Liu et al., 2020). Additionally, spectral analysis post-blink latency has shown early power increase in delta and beta/low-gamma frequency bands (12–35 Hz) representing the response to visual processing caused by blinking, followed by a later and longer power decrease in alpha (7–13 Hz) and theta (4–7 Hz) band which has been attributed to information processing and episodic memory, respectively (Liu et al., 2019b).

Given the potential link between changes in the features of the EEG signal, alterations in DMN function following mTBI, and the implication of precuneus in BRO response, a key hub of default mode network (Hagmann et al., 2008), we hypothesized that the study of the relationship between this response and subconcussive head impacts in athletes could provide valuable insights into the neural mechanisms underlying the effect of such impacts. It is important to note that, while the incidence and impact of concussion and subconcussion varies between sexes, very few studies specifically address female athletes (Koerte et al., 2020). Therefore, our study is specifically focused on female soccer players in order to address this key gap in the literature.

Our aim was to evaluate the response features of the BRO as a potential brain-based biomarker in a cohort of female varsity soccer athletes compared with a control group of healthy, female varsity athletes involved in non-contact sports. We predicted that changes in BRO responses would be associated with subconcussive impacts.

## 2. Materials and methods

### 2.1. Data collection and pre-processing

#### 2.1.1. Participants

At the beginning of this study, the research team (PI and graduate students) made a presentation to The University of British Columbia’s women’s soccer team and their coaches. The background and purpose of the study was discussed and volunteers who wished to take part were asked to contact a member of the research team. Thirteen players volunteered for the study and 10 players who met the eligibility criteria were recruited at the end of their soccer season (SC group). The eligibility criteria included (1) age 18–25 years and (2) active participation in soccer throughout the season. Participants who identified any health related comorbidities (e.g., diabetes, thyroid, hypertension, etc.) were not eligible to participate. The head coach identified the players who were active in using their head during practices and games. The physical therapists and team physician reported that none of the participants had a diagnosed concussion during the entire season and none of the participants were experiencing any symptoms of concussion at the time of testing. Ten healthy female varsity athletes were recruited from a pool of female varsity athletes who had previously volunteered to take part in research studies conducted at The University of British Columbia. All control athletes reported that they had no history of concussion nor any history of sub-concussive impacts and participated in varsity sports that do not involve heading, such as swimming and rowing (HC group). Participants reported that they had a normal or corrected-to-normal vision. A total of four participants reported corrected vision. The study was approved by the Human Ethics Review Board of The University of British Columbia (UBC REB H17-02973) and all players provided informed consent to participate. The study took place during the summer soccer from May 2019 until August 2021. All data collection took place at the end of the summer session.

#### 2.1.2. EEG recording

Electroencephalography was recorded from each participant in a wakeful rest condition over a period of 5 min using a 64-channel EEG system (EGI, Eugene Oregon) placed according to the 10–20 system, with electrode impedances kept below 50 kΩ. The reference was the vertex (Cz), and the sampling rate was 250 Hz. In an experimental room with consistent light levels, participants were instructed to maintain a constant gaze at a white board in front of them and to refrain from moving. The entire EEG recording session took between 30 and 45 min per participant.

#### 2.1.3. EEG pre-processing

**FIGURE 1:**
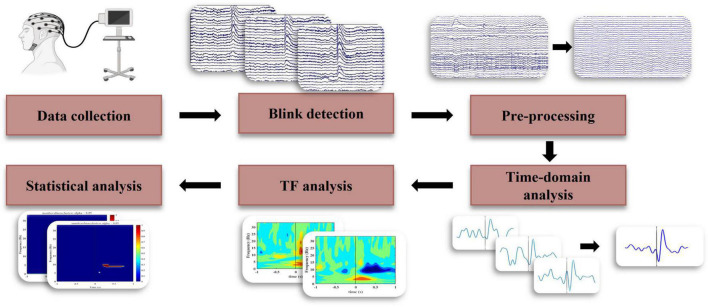
Block diagram for EEG data analysis to identify the temporal and spectral characteristics of BRO response.

The EEG data was first re-referenced to the average of all channels. Following this, the data was notch filtered at 60 Hz, and bandpass filtered from 0.5 to 80 Hz using a 4th-order Butterworth filter with zero phase shift. Independent component analysis, using the runica algorithm (Delorme and Makeig, 2004), was performed to remove artifactual components by considering the time, topographic, and power distribution of components. Pre-processing was done using EEGLAB (ver. 2021.1) in the Matlab (ver. R2021a) environment. Figure 1 shows the steps of the entire process from data collection to final analyses.

#### 2.1.4. Blink detection

In order to extract blink latencies, raw vertical electrooculogram data was first filtered from 0.5 to 20 Hz and passed to the EEGLAB function BLINKER (Kleifges et al., 2017). The result from this analysis was evaluated and corrected through manual inspection. A temporal threshold of three seconds was applied to blink latencies to minimize the contamination of epochs by extracting only blinks that lay 3 s apart from their adjacent blinks aligned with previous studies (Liu et al., 2017, 2019b, 2020). After temporal thresholding, participants with fewer than five blink epochs were excluded from the study. A total of 9 participants in the SC group and 10 participants in the HC group passed this exclusion criterion. The pre-processed data was segmented based on the final latencies to create 3-s epochs from −1.5 s before and 1.5 s after the blink latencies (Figure 1).

**FIGURE 2:**
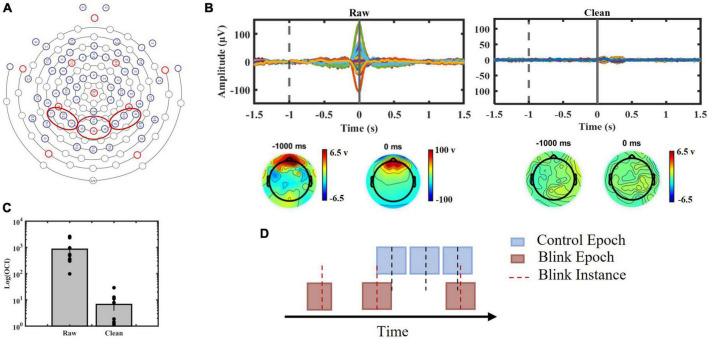
**(A)** EGI 64-channel sensor layout used in the study with red circles indicating channels of interest. Channels 27, 28, and 31 were chosen to represent left parietal region, channels 34, 35, 36, and 38 were chosen to represent central parietal region and channels 40, 42, and 45 were chosen to represent right parietal region. **(B)** Top panels: Blink epochs, 64 channels presented before (Left) and after (Right) ICA procedure. Bottom panels: topographic maps presenting channel voltages at –1000 ms (baseline) and 0 ms (blink peak time) before (Left) and after (Right) artifact removal. **(C)** Blink-to-baseline power ratios before and after artifact rejection (*p* < 0.01). **(D)** Schematic illustration of data segmentation procedure.

To control for background brain activity, control epochs were randomly generated, consisting of 3-s consecutive epochs with the same number as BRO epochs for each subject. These control epochs were not time-locked to the latencies of blinks (Figure 2D). They were subsequently used to compare background brain activity with BRO features and to validate the association of these features with post-blink processing (Supplementary material).

For the purpose of analyzing behavioral differences, the blink rate was calculated for each participant by dividing the number of blinks detected in the data (before temporal thresholding) by the length of the data in minutes. Blink rates of the two groups (SC and HC) were compared using two-tailed, independent *t*-test.

#### 2.1.5. Effectiveness of artifact removal

To ensure the efficacy of the ocular artifact removal, the trial averaged blink epochs before and after ICA was manually inspected. Time-domain blink-response of all channels along with topographic maps were observed at the time of blinking (0 ms) and baseline (−1000 ms), which was selected to ensure separation from potential blink-related saccadic eye movement. The removal of large spikes related to ocular movement was examined (Figure 2B). Furthermore, we computed the ocular contamination index (OCI) for each participant to quantify the blink-to-baseline signal power before and after artifact removal (Figure 2C). OCI was computed using the following formula align with previous studies (Liu et al., 2017, 2019b, 2020):


$$O\,C\,I=\frac{\sum_{i=1}^{n}{\left(y_{i}-\bar{y}\right)}^{2}}{\sum_{i=1}^{n}{\left(x_{i}-\bar{x}\right)}^{2}}$$

Where n is the number of channels, *y*_*i*_ and *x*_*i*_ represent signal amplitude at 0 and −1000 ms, respectively, corrected for channel drift by subtracting the average amplitude over all channels.

Two-tailed, paired *t*-test was performed on raw and cleaned data.

#### 2.1.6. Time domain analysis

Three areas corresponding to the parietal region were selected for analysis (Figure 2A) as BROs were initially detected in this area (Liu et al., 2017). Data from the selected channels were first filtered in delta band (0.5–4 Hz) using a 4th order Butterworth filter with zero phase shift and then segmented into blink epochs (Liu et al., 2020). The average amplitude within an interval of −1.5 to −0.5 s was subtracted as the pre-blink baseline. Following the calculation of the average of all trials for each individual, the average response of channels of interest was calculated for each selected region.

For each participant, the amplitude of the largest negative peak and its corresponding time within the 50–200 ms window post-blink, as well as the amplitude of the largest positive peak in the 250–400 ms window post-blink, were measured.

Pearson correlation was used to investigate the amplitude and latency relationships with number of headers in SC group. A two-tailed, independent *t*-test was used to compare peak amplitude and latency between the two groups (SC vs. HC). The significance threshold was adjusted using a Bonferroni’s correction method.

#### 2.1.7. Spectral analysis

The Stockwell transform was employed (Stockwell et al., 1996) to obtain the power spectrum. From the complex matrix of the transform at each time point absolute value was calculated. Time window of −1.5 to −0.5 s was selected to establish a baseline and average power of the baseline was subtracted from the power spectrum of each epoch. Lastly, the average power across epochs was calculated for each subject, and the grand average of each group was calculated.

Cluster-based permutation statistics were performed to compare the power spectra of BRO and control trials and to compare the power spectra of the BROs between the two groups (Maris and Oostenveld, 2007; Liu et al., 2019a; Hajra et al., 2020). To do this, a *t*-test was first performed at each frequency point throughout the epoch time and the maximum cluster size exceeding the significance threshold was identified. Data from the two groups was then permuted 1000 times, achieved by scrambling and redistributing the data into new groups randomly while maintaining the sample size. The same *t*-test procedure was performed on each permutation with corrected threshold for multiple comparison. The final error rate was calculated by determining the number of greater cluster sizes observed after permutation compared with the original maximum clusters. Clusters with an error rate lower than 0.05 were considered statistically significant and were used to identify features of the spectrum that differed significantly between conditions or groups.

Furthermore, correlation analyses were performed between the number of head impacts sustained during a season of play and the spectral features of the BRO response. Distinct spectral features for each frequency band were specifically focused on, and three sets of correlations were conducted (one for each region). However, due to the interdependence of the three brain regions arising from volume conduction, the Holm-Bonferroni’s correction (Holm, 1979) was applied to address the issue of multiple comparisons.

#### 2.1.8. Video analysis

All on-field activity was video recorded during one season. Videos were watched at normal speed to identify impacts, and each impact was reviewed in slow motion or frame-by-frame for further characterization. An athlete exposure was defined as any practice, scrimmage, or game event that an athlete was involved in. A subconcussive head impact was defined as an observed ball to head impact. Details of the methods are provided in Kenny et al. (2022). The cumulative number of headers for each player was calculated. Considering the potential of greater head exposure during practice, we also included practice time in our analysis (Kenny et al., 2022). The cumulative number of headers for each participant at the end of the season was used in comparison analyses to identify relationships between numbers of headers and BRO features.

## 3. Results

### 3.1. Participant demographics

Table 1 shows the demographic information of the participants in the study. The healthy control (HC) group self-reported that they had no previous concussions nor any headers exposure and participated in non-contact varsity sports. The subconcussive (SC) group was sampled at the end of the season and the headers during the season were calculated based on video analysis.

**TABLE 1:** Demographic information of participants.

	HC	SC
Total *N*	10	10
Age in years (mean ± SD, range)*	22.6 ± 1.9 (19–25)	19.7 ± 1.4 (18–25)
Height in meters (mean ± SD, range)	1.7 ± 0.1 (1.5–1.8)	1.7 ± 0.1 (1.6–1.8)
Weight in kilograms (mean ± SD, range)	64.1 ± 9.3 (49–82)	64.9 ± 4.9 (59–72)
Length of playing career in years (mean ± SD, range)*	9.4 ± 4.2 (5–15)	15.2 ± 1.3 (10–17)
Years of heading (mean ± SD, range)*	0	10.1 ± 2.2 (7–14)
Headers during the one season (mean ± SD, range)*	0	195.4 ± 34.7 (141–235)

*Denotes statistically significant differences (*p* < 0.05). SC, subconcussive cohort; HC, healthy control cohort.

### 3.2. Blink rates

The SC group exhibited a higher number of blinks per minute compared to HC group.

[SC (#/min): 16.12 ± 8.13, HC (#/min): 10.07 ± 6.09], however, the difference was not statistically significant (*p* = 0.09).

### 3.3. Artifact removal

The evaluation of the effectiveness of ocular artifact removal demonstrated the successful elimination of the large peak associated with eyelid movement at 0 ms (Figure 2B). Furthermore, a comparison of OCI (Figure 2C) exhibited a significant reduction in the contribution of blink ocular artifact pre- and post-application of ICA analysis (*p* < 0.01).

### 3.4. Time domain results

**FIGURE 3:**
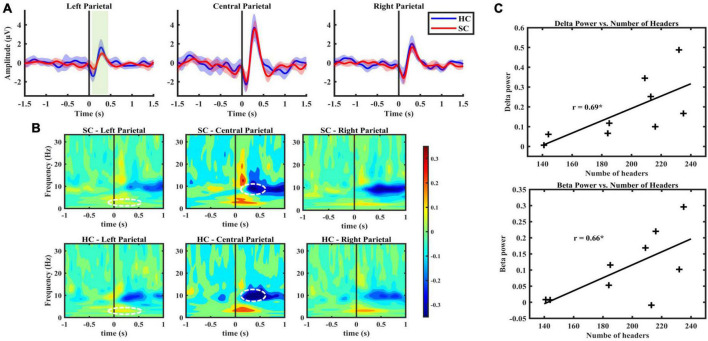
**(A)** BRO response, grand averaged over all subjects with the narrow shading indicates 95% confidence interval, indicating low variability between subject responses. From left to right presents the average response from the left, central and right parietal channels, respectively. **(B)** Figure illustrates spectral characteristics of BRO response with time zero indicating blink latencies. The color bar indicates power derived from Stockwell transform values. SC group exhibited lower decreases in alpha power compared with baseline in central parietal, as well as a lower increase in delta power in the left parietal region compared to HC group (*p* < 0.05). **(C)** A significant positive correlation observed in central parietal region between BRO features in frequency domain and the number of headers in SC group.

Blink-related oscillation responses were detected in both SC and HC groups, predominantly in central (33, 34, 36, 38) and right (40, 42, 45) channel clusters. The morphological characteristics were found to be consistent with previous literature of BRO (Liu et al., 2019b, 2020). These characteristics include a first negative peak occurring within a 50–200 ms latency window followed by a positive peak occurring within a latency window of 250–400 ms (Table 2). Notably, a left lateralized effect was observed in the left parietal region, indicating a statistically significant decrease in the amplitude of the first peak in the SC group compared to HC (*p* < 0.05, Cohen’s d = 0.03), and a lower peak-to-peak value, respectively, (*p* < 0.05, Cohen’s d = 0.14) (Figure 3A).

**TABLE 2:** Morphological characteristics of the BRO response.

Region		Negative peak amplitude (μ V)	Negative peak latency (ms)	Positive peak amplitude (μ V)	Positive peak latency (ms)	Peak-to-peak amplitude (μ V)
Left	SC	−0.91 ± 0.78	97.33 ± 58.99	1.15 ± 0.42	303.55 ± 45.05	2.06 ± 0.82
	HC	−1.74 ± 0.98*	91.20 ± 45.01	1.92 ± 1.28	297.60 ± 59.87	3.67 ± 2.01*
Center	SC	−2.1 ± 1.53	86.22 ± 31.31	3.76 ± 1.54	292.44 ± 30.62	5.89 ± 2.84
	HC	−2.79 ± 1.68	101.20 ± 38.55	4.12 ± 2.53	289.60 ± 33.42	6.09 ± 3.81
Right	SC	−1.82 ± 1.06	98.66 ± 39.14	1.87 ± 1.04	304.88 ± 32.50	3.69 ± 1.93
	HC	−1.83 ± 1.57	88.40 ± 57.08	2.19 ± 1.56	306.80 ± 32.49	4.03 ± 2.90

SC, subconcussive group; HC, healthy control group. *Illustrates *p* < 0.05 between HC and SC.

Table 2 presents the morphological characteristics of the BRO response, including peak amplitude and latency. The measures were calculated individually and are reported as mean ± standard deviation.

### 3.5. Frequency domain results

The results of the frequency domain analysis revealed BRO-related alterations in spectral characteristics following blink response in both SC and HC groups across all three channel clusters (Figure 3B). The observed changes comprised an increase in delta (0.5–4 Hz) band power lasting approximately 0.5 s after the blink, followed by a decrease in alpha (7–13 Hz) band power initiated 0.25 s post-blink latency, as well as an increase in lower beta (13–20 Hz) band power following the blink response. The use of cluster-based permutation statistics for the comparison of blink epochs and control epochs revealed these spectral features aligned with previously reported studies on BROs (Liu et al., 2017, 2019b; Supplementary material). This comparison further confirms the distinction between spectral changes post-blink latency and brain background oscillatory activity. Moreover, it provides evidence for the association between these spectral changes and blink-related processing activity in the brain.

Lastly, significant differences were observed between SC and HC groups concerning the central and left channel clusters’ spectral changes (*p* < 0.05). These differences include diminished post-blink power changes in the alpha band relative to the baseline for the SC group with the effect size of 1.2 reported based on the maximum effect within the cluster identified to be significantly different, as well as reduced increase in delta power in the left parietal region for the SC group with the maximum effect size of 0.8 within the cluster (Figure 3B).

### 3.6. Association between number of headers and BRO features

Correlation analysis investigated the relationship between the number of headers sustained during a season of play and the BRO features in both time and frequency domains. A significant positive association was found between the immediate post-blink latency beta and delta power and the number of headers, indicating that higher beta and delta band power were correlated with a greater number of headers sustained during the season (Figure 3C).

## 4. Discussion

### 4.1. Main findings

This is the first study to explore the potential of blink-related oscillations as an indicator of subtle neural changes caused by subconcussive head impacts. In the present study, we investigated the average regional BRO responses over the left, central, and right parietal cortices, where the response was initially identified (Bonfiglio et al., 2013; Liu et al., 2017). We collected 5 min eyes-open resting state EEG data from two cohorts of female collegiate athletes involved in contact vs. non-contact sports, as per our previous published studies (Virji-Babul et al., 2014; Liu et al., 2020). The results revealed that time-domain delta BRO response morphologies were characterized by an initial negative peak occurring in the 50–200 ms window, followed by a subsequent positive peak in the 250–400 ms window post-blink latency. Narrow 95% confidence interval indicated low variability of response morphologies between individuals. We also examined spectral characteristics of BROs which demonstrated a blink-related increase in delta band power immediately after blink latency up to 400 ms post-blink, followed by a decrease in theta/alpha band power at 200 ms post-blink compared to pre-blink baseline.

The primary findings of our study regarding the differences between the two investigated groups of athletes are as follows:

Firstly, the peak-to-peak amplitude of response in the channels over the left parietal region was significantly decreased in the SC group as compared to the HC group. Secondly, there were significant differences in BRO spectral power in the delta and alpha frequency range in the SC group compared to the HC group. Over the left parietal channels, the delta power of the SC group was reduced. Furthermore, the central parietal channels exhibit significant differences in spectral power in high theta/low alpha frequencies.

Importantly, there was a robust linear relationship between the increase in power over central parietal channels in the delta and beta frequency ranges and the number of headers sustained by the SC group during a single season of play and practice.

### 4.2. Interpretation of findings

Although there is a growing body of literature on the cumulative effect of subconcussive head impacts, there is still a lack of objective assessments of the subtle brain changes resulting from these types of head impacts. A number of studies have demonstrated a relationship between the cumulative effect of head exposure and the development of cognitive, memory, and neurodegenerative disorders (Ntikas et al., 2022). However, these changes typically cannot be detected until the cumulative effects of the impacts have reached the point where specific diagnoses are possible. To better understand the origin of these alterations, it is essential to identify subtle microstructural and functional changes caused by head impacts at an early stage. The advantages of EEG-based assessment lie in the ease of use in point-of-care settings such as sports arenas and the ability to frequently monitor changes over time. Blink-related EEG oscillations provide valuable information about the resting-state brain without the need for extensive testing or very specialized equipment or sensory input required for evoked response. This approach holds promise for evaluating neural oscillatory changes due to subconcussive head impacts. This study serves as an initial proof of concept, demonstrating that changes in BRO features are associated with repetitive head impacts in contact sports.

Slower oscillations are known to modulate faster, more localized oscillations involved in higher-order processing. Delta oscillations have been linked to inhibitory function during internal concentration and attention (Harmony, 2013; Simis et al., 2022). The immediate increase in delta band power following the blink latency may be related to the brain’s effort to suppress irrelevant sensory inputs and focus on processing the image that appeared after the eyelid is re-opened. Furthermore, intrinsic lower beta rhythms have been shown to be associated with maintaining spatiotemporal neural priors (Betti et al., 2021). The increase in lower-beta power post-blink latency further confirms the brain’s effort to understand the environment based on recent history (Betti et al., 2021). The association between the increasing number of headings and increased delta and beta power in the SC group may be a result of compensatory brain mechanisms associated with inhibition and prediction required for processing post-blink images. This association suggests that as the number of head impacts increases, the internal processing of spontaneous blinking may become more energy-intensive for the brain in the SC group.

Another prominent feature of the BRO response is the decrease in alpha power ∼ 200 ms post-blink latency. The relationship between decreased alpha power and working memory, as well as the active state of visual processing is well established in the literature (Wianda and Ross, 2019). This association further supports the brain effort for processing of post-blink image. In the comparison between SC and HC group, we observed a significant decrease in alpha power for HC group. This may be due to the SC group’s inability to effectively suppress alpha oscillations to perform the visual processing task. Importantly, previous studies have shown a connection between a greater decrease in alpha oscillation power and better working memory performance (Erickson et al., 2019; Wianda and Ross, 2019). These findings are consistent with recent studies on experienced professional soccer players, which reveal abnormal reduction in cortical thickness in bilateral parieto-occipital region, known to play a role in visuospatial functioning, visual working memory and attention (Koerte et al., 2016, 2020; Strauss et al., 2021), which aligns with the result of our study. This is an important distinction and highlights the importance of further investigations into working memory performance and its association with subconcussion.

The time-domain delta oscillation results indicated that the oscillation is phase-locked to the time of spontaneous blinks, meaning that the process of trial averaging would diminish the peak amplitudes if the oscillations were not synchronized with blink time. The reduced amplitude of the peak-to-peak value of delta oscillations in left channel clusters in SC group might suggest a disruption in the synchrony between oscillation timing and spontaneous blinking or a reduced neural resource for processing the response. Regardless of the cause, this difference could reflect the subtle microstructural changes in the brain resulting from repetitive head impacts. These findings are consistent with existing literature that reports microstructural changes and alterations in white matter integrity as a result of subconcussions, predominantly in the areas of corpus callosum, the internal capsule, the fornix, the cingulum bundle, and the superior longitudinal fasciculus (Abbas et al., 2015; Koerte et al., 2015).

it’s important to note that mTBI is known to impact the dopaminergic systems of the brain (Chen et al., 2017; Kulkarni et al., 2019). There is also a well-established link between dopamine and blink rates to the point of blinks being considered a non-invasive marker of dopamine levels (Jongkees and Colzato, 2016). In our study, we did not find significant differences in blink rate between athletes involved in contact sports and those in non-contact sports; however, the association with dopamine could imply subtle neurological changes that modulate other observed differences in blink-related oscillations and related EEG features. A recent study using MEG has shown that BROs can detect changes in normal aging (Liu et al., 2021) suggesting that this response may be a very useful and powerful tool for detecting the earliest changes in brain responses in a variety of conditions.

### 4.3. Potential future direction: incorporation AI into diagnostic and prognostic models

Building on the preliminary results from our study, future work will benefit from the application of artificial intelligence (AI) to classify subconcussive and concussive impacts. AI is currently being leveraged using EEG data in monitoring and prognosis of head impacts (Hajra et al., 2019; Thanjavur et al., 2021). This study could potentially contribute to enhancing the predictive value of a brain-based biomarker for detection of subtle neural changes due to repetitive head impacts along with incorporation of other potential contributing factors such as headache, visual disturbances and mental health issues.

### 4.4. Limitations

This study has several limitations. First, we acknowledge that the small sample size and cross-sectional design limit the generalizability of the findings. A larger and more diverse sample including male data would strengthen the validity of the results, allow for more accurate identification of potential biomarkers and provide an analysis of sex-based differences. Additionally, a larger control population would help in establishing a gold standard for the BRO response and more effectively identify abnormalities in the SC group. This would enhance the clinical utility of the observed biomarker. Another limitation in the current study methodology is the lack of recognized identification method of TBI history. To determine the long-term effects of heading in soccer on brain health, longitudinal studies with larger sample sizes are clearly needed. Future studies should collect information about the head and neck measurements and regress these effects as these features have been shown to have an effect on the prevention of concussion injuries (Cooney et al., 2022). It should be noted that blink rates can be affected by diurnal variations and the use of birth control. In our cohorts, data was collected at the same time and there were no significant differences in the use of birth control. To date, no significant correlation of contact lens use, temperature, humidity, age or menstrual cycle have been found for blink rates.

## 5. Conclusion

We report a strong association between the number of head impacts in female collegiate soccer players through one season of play with spectral delta and beta band power increase in a resting state condition following blinking. We found several time related changes as well as changes in the spectral morphology of the BRO response in the SC group. This preliminary investigations suggests that the BRO response may be a useful, cost-effective brain-based biomarker for the detection of subtle neural changes due to repetitive head impacts. Larger sample sizes with a more diverse demographic as well as follow up studies are needed to validate the utilization of the response as a potential biomarker. Further detailed studies on the relationship between cognition and motor behavior as a function of changes in the BRO response may also inform the nature of long-term impacts. These studies will create a baseline to establish injury prevention protocols and to enhance player performance.

## Data availability statement

The datasets presented in this article are not readily available because the Human Ethics application would have to be changed to request permission from the participants before the data can be made available. Requests to access the datasets should be directed to NV-B, nvb31@mail.ubc.ca.

## Ethics statement

The studies involving human participants were reviewed and approved by Human Ethics Review Board of The University of British Columbia (UBC REB H17-02973). The patients/participants provided their written informed consent to participate in this study.

## Author contributions

SS, CL, SH, and NV-B contributed to conception, design of the study, and overall design of the manuscript. RK and SS collected the data. SS pre-processed, prepared the data, and wrote the first draft of the manuscript. SS, CL, SH, and GD contributed to the analysis of the data and statistical analysis. CL, SH, NV-B, and GD contributed to the manuscript revision. All authors read, and approved the submitted version.
