# Supplementary material for: Blink-related EEG oscillations are neurophysiological indicators of subconcussive head impacts in female soccer players: a preliminary study
Source: Front Hum Neurosci. 2023 Jul 19;17:1208498. doi: 10.3389/fnhum.2023.1208498 (PMC10394644; doi:10.3389/fnhum.2023.1208498)
Supplement: Supplementary file 1 [file Data_Sheet_1.docx]

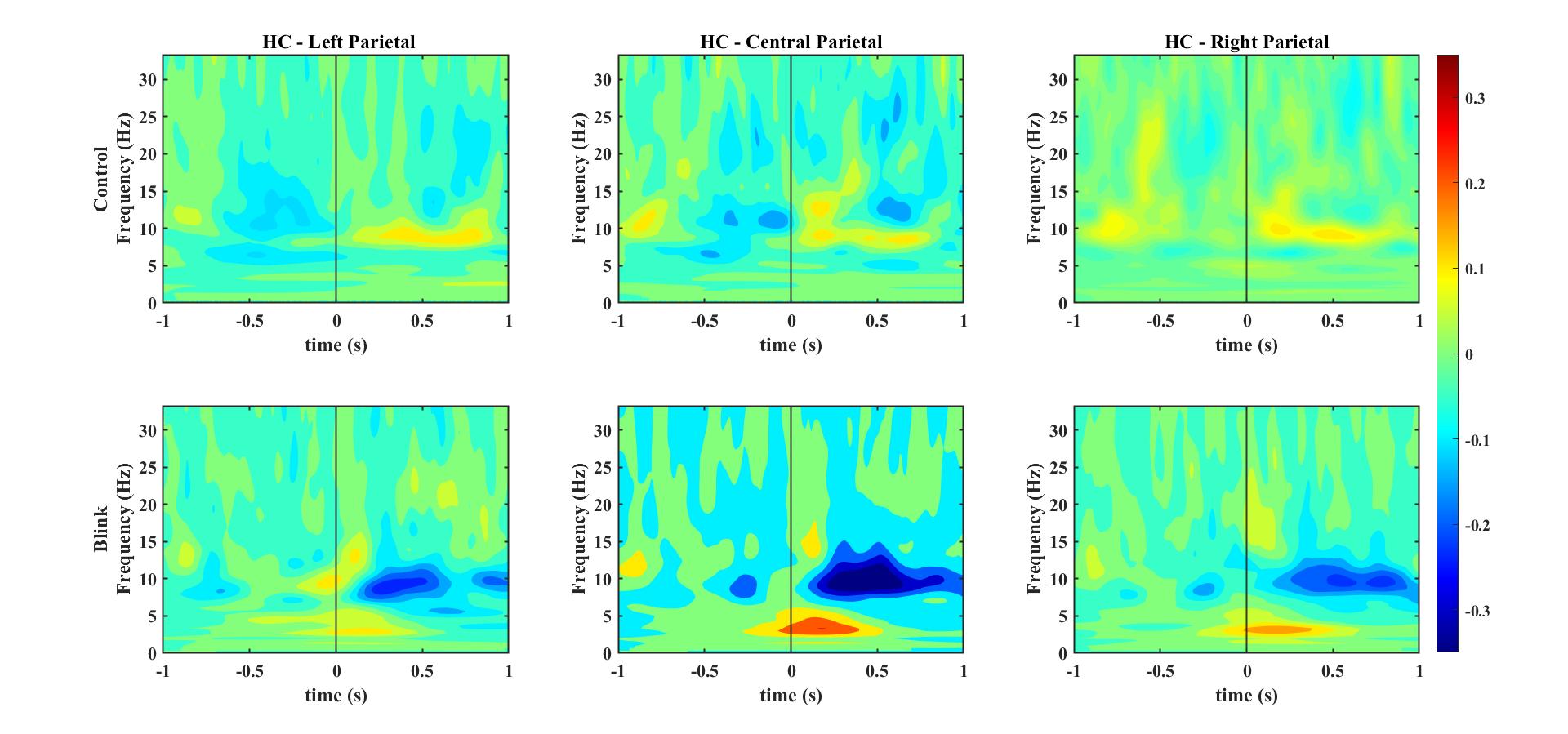


B

A


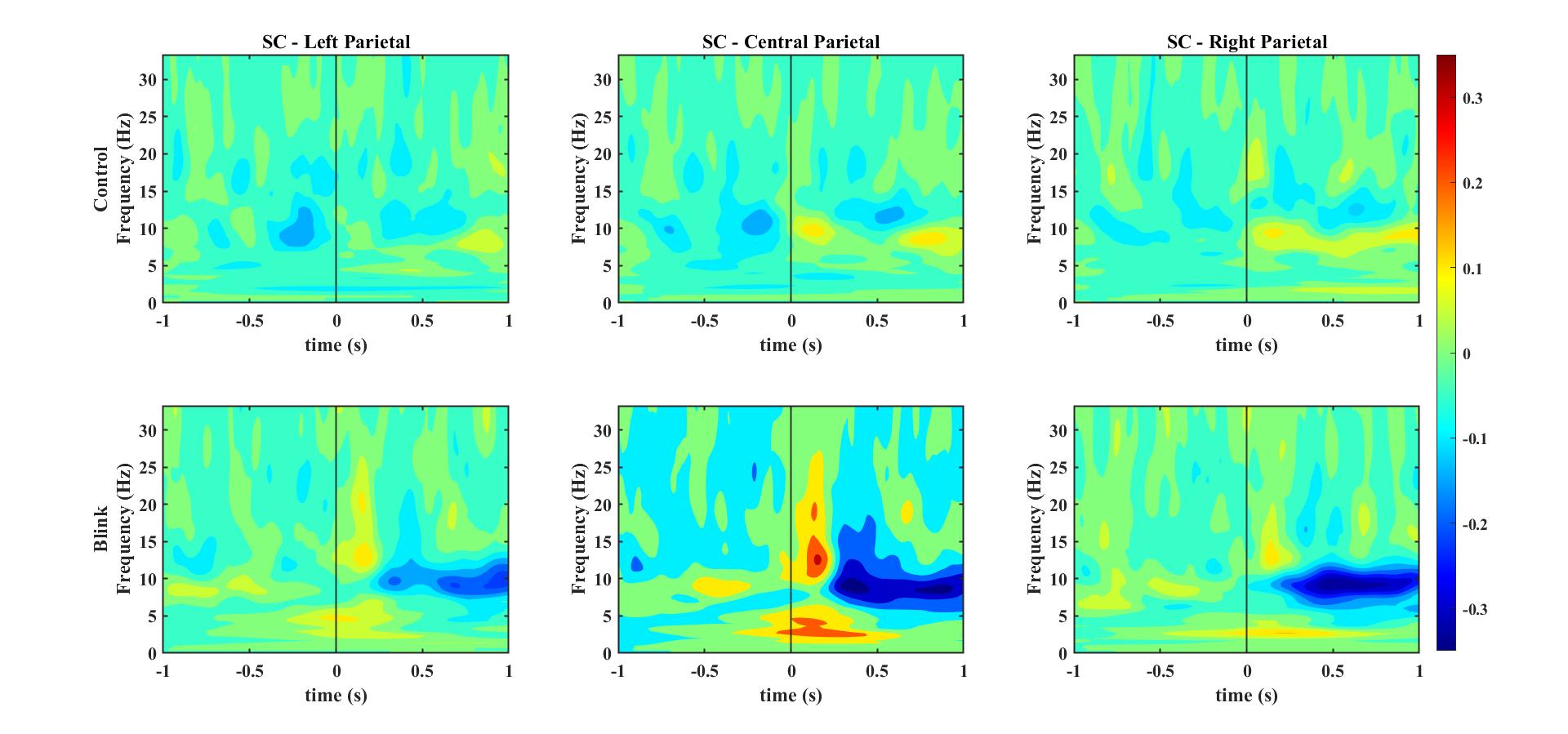


Figure S1: Temporal evolution of blink-related spectra; features is evident in the “Blink” condition, while absent in the “Control” condition (P<0.05) due to the lack of time-locking to blink occurrences for both HC (A) and SC (B) groups.
